# A machine learning model for non-invasive detection of atherosclerotic coronary artery aneurysm

**DOI:** 10.1007/s11548-022-02725-w

**Published:** 2022-08-10

**Authors:** Ali A. Rostam-Alilou, Marziyeh Safari, Hamid R. Jarrah, Ali Zolfagharian, Mahdi Bodaghi

**Affiliations:** 1 Department of Engineering, School of Science and Technology, Nottingham Trent University, Nottingham, NG11 8NS UK; 2 School of Engineering, Deakin University, Geelong, 3216 Australia

**Keywords:** Atherosclerosis, Coronary artery aneurysm, Machine learning, Computed tomography angiography, Non-invasive detection, Hemodynamics, Morphometry

## Abstract

**Purpose:**

Atherosclerosis plays a significant role in the initiation of coronary artery aneurysms (CAA). Although the treatment options for this kind of vascular disease are developing, there are challenges and limitations in both selecting and applying sufficient medical solutions. For surgical interventions, that are novel therapies, non-invasive specific patient-based studies could lead to obtaining more promising results. Despite medical and pathological tests, these pre-surgical investigations require special biomedical and computer-aided engineering techniques. In this study, a machine learning (ML) model is proposed for the non-invasive detection of atherosclerotic CAA for the first time.

**Methods:**

The database for study was collected from hemodynamic analysis and computed tomography angiography (CTA) of 80 CAAs from 61 patients, approved by the Institutional Review Board (IRB). The proposed ML model is formulated for learning by a one-class support vector machine (1SVM) that is a field of ML to provide techniques for outlier and anomaly detection.

**Results:**

The applied ML algorithms yield reasonable results with high and significant accuracy in designing a procedure for the non-invasive diagnosis of atherosclerotic aneurysms. This proposed method could be employed as a unique artificial intelligence (AI) tool for assurance in clinical decision-making procedures for surgical intervention treatment methods in the future.

**Conclusions:**

The non-invasive diagnosis of the atherosclerotic CAAs, which is one of the vital factors in the accomplishment of endovascular surgeries, is important due to some clinical decisions. Although there is no accurate tool for managing this kind of diagnosis, an ML model that can decrease the probability of endovascular surgical failures, death risk, and post-operational complications is proposed in this study. The model is able to increase the clinical decision accuracy for low-risk selection of treatment options.

## Introduction

An aneurysm arises when a blood vessel is weakened due to the influence of the combination of several causes such as atherosclerosis (arterial wall hardening), aging, genetics, and other physiological and environmental factors leading to a bulge initiation in the arterial wall tissue. This may happen in any blood vessel in the human body, especially in the branches. However, two main classifications of aneurysms are: the abdominal aorta aneurysm and the brain aneurysm. Besides these, the Coronary Artery Aneurysm (CAA) is one of the rare cardiovascular diseases with a 1.2%–4.9% occurrence rate [1] associated with a variety of factors, coronary atherosclerosis being one of them [2]. In fact, the first etiology of CAAs is atherosclerosis and arterial occlusion. This kind of aneurysm may appear in different phenotypes of saccular, fusiform, and ectasia.

**Fig. 1:**
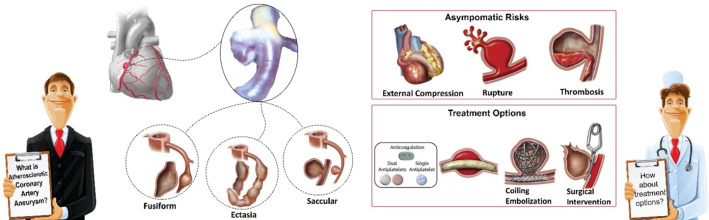
Schematic view of an atherosclerotic coronary artery aneurysm, its phenotypes, and treatment options

Based on the aneurysmal nature, the CAAs are also silent and asymptomatic, causing thrombosis, aneurysm rupture, and external compression on the myocardium. On the other hand, the management of CAAs is faced with some challenges like variable definitions, often asymptomatic, limited outcome data, and no specific guidelines. In some cases, conservative treatment may be applied with medical control and management, but the other treatment options such as surgical methods for excision or bypass grafting, covered stent, coiling, clipping (surgical intervention) [3], and smart biomaterial-based methods [4] are also advanced alternatives. Figure 1 illustrates a brief graphical overview of coronary artery aneurysm and treatment options. In comparison with cerebral atherosclerotic aneurysm, studies are relatively few to reveal more aspects of CAAs. Therefore, paying more attention to the hemodynamics and morphology of CAAs plays a significant role in early diagnosis and applying successful treatments. In recent decades, biomechanical study techniques of vascular diseases have been developing not only in computed tomography angiography (CTA) investigations but also in numerical and computational mechanics with engineering simulations. On the other hand, the development of non-invasive predictors based on modern CTA used methods [5] for coronary artery diseases is attracted significant attention from scientists. For example, Weber et al. [6] used a series of CTAs for coronary plaque volume assessment and design of predictors to non-invasive detection of fast plaque progression. Some researchers have tried to use other imaging tools such as magnetic resonance angiography to increase the enhancement of contrast-enhanced for early detection of coronary atherosclerotic plaque in patients [7]. Besides, the use and design of artificial intelligence tools for obtaining faster and more accurate results in numerical, visual, and other aspects of biological process investigation is expanding by scientists. The potential use of AI in the prediction of abdominal aortic aneurysm expansion is investigated by Jiang et al. [8] using a deep learning algorithm with longitudinal data that is based on repeating observations related to the same variables over long or short periods of time. Similar studies were performed by Liang et al. [9] to investigate the relationship between morphometric properties and numerically predicted risk of aortic aneurysm growth applying an ML algorithm. Most of the performed studies related to using AI in aneurysms are supported by data sets from kinds of studying methods such as in-vivo clinical experiments [10], segmentation 1-dimensional flow [11, 12], the DL techniques based on in silico pressure [13], and CTA-based datasets. However, some studies are focused on explaining growth [14], automatic detection according to morphometric features [15–17] and early detection [10] of aortic aneurysms. Although some researchers have proven the efficiency of the ML models for use in vascular diseases associated with atherosclerosis, the CAAs have received little attention from scientists because of database limitations. For example, [18, 19] and [20] proposed novel medical diagnosis support systems for predicting patients with atherosclerosis diseases using ML algorithms. The ability of the ML to demonstrate the importance of hemodynamics for studying atherosclerotic coronary arteries in the presence of stenosis was performed by Farajtabar et al. [21]. In addition, Alizadehsani et al. [22] employed ML algorithms to design a model for non-invasive detection of coronary artery diseases using the prediction of stenosis in coronary arteries separately. The most recent overview literature is available at [23] that focuses on current applications of ML for understanding atherosclerosis and plaque formation. Also, a comprehensive review of advanced imaging techniques for atherosclerosis by CTA supporting Radiomics and learning approaches is presented by Kolossvary et al. [24] An ML-based tool for CTA evaluation of coronary arteries involved in atherosclerosis is proposed by Choi eta l. [25] considering stenosis and vascular morphology. Their model was able to identify a wide range of plaque volume and composition in the corresponding coronary artery and its branches. The impact of stenosis location on the coronary artery was also investigated by Renker et al. [26] to show how an ML-based model can work with CTA and invasive fractional flow reserve for analysis of large and multicenter datasets.

Although endovascular therapies are young methods for both cerebral and coronary artery aneurysms treatment, they are also faced with limitations that are under advanced investigation to be solved. A challenging problem that arises in the clipping method is the difficulty in the placement of clips on the dome or neck of the aneurysm close to the atherosclerotic plaque aggregation zone. Despite trying some ideal alternative contrivances, the outcomes are usually unfavorable [27]. However, making a suitable decision to select the most appropriate endovascular method for the patient requires more patient-specific data before surgical interventions. Coronary CTA, on the other hand, is able to present non-invasive visualization of the coronary lumen, arterial wall, and coronary occluded by stenoses [28], but it cannot detect aggregated plaques nearby the aneurysm. To this end, employment of an AI model that is able to diagnose the atherosclerotic aneurysm from the non-atherosclerotic one, using morphological and hemodynamic features of aneurysms to perform, will be a non-invasive clinically applicable tool. In this study, the authors propose an ML model for the detection of atherosclerotic CAAs that can be a unique AI tool for collecting non-invasive tests for assurance in decision-making procedures of applying suitable endovascular methods. All confirmed datasets relevant to the atherosclerotic CAAs are collected from the work presented by Fan et al. [29] that was performed in order to study morphometric and hemodynamic analyses in the epicardial coronary arteries of patients with CAAs caused by atherosclerosis. Then, the ML algorithms are developed to design an optimum model with accurate outputs according to the dataset. In section “History of database”, the employed learning approach is presented. The obtained results and discussion on the eligibility of the model for utilization as an AI tool for non-invasive detection of atherosclerotic CAAs are also explained in section “Results and discussion on ML model feasibility”. Due to the absence of similar ML model and results in the specialized literature, this paper is likely to fill a gap in the state of the art this problem and provide pertinent results that would be instrumental for assurance in clinical decision-making procedures for surgical intervention treatment methods.

## Materials and machine learning approach

### History of database

**Fig. 2:**
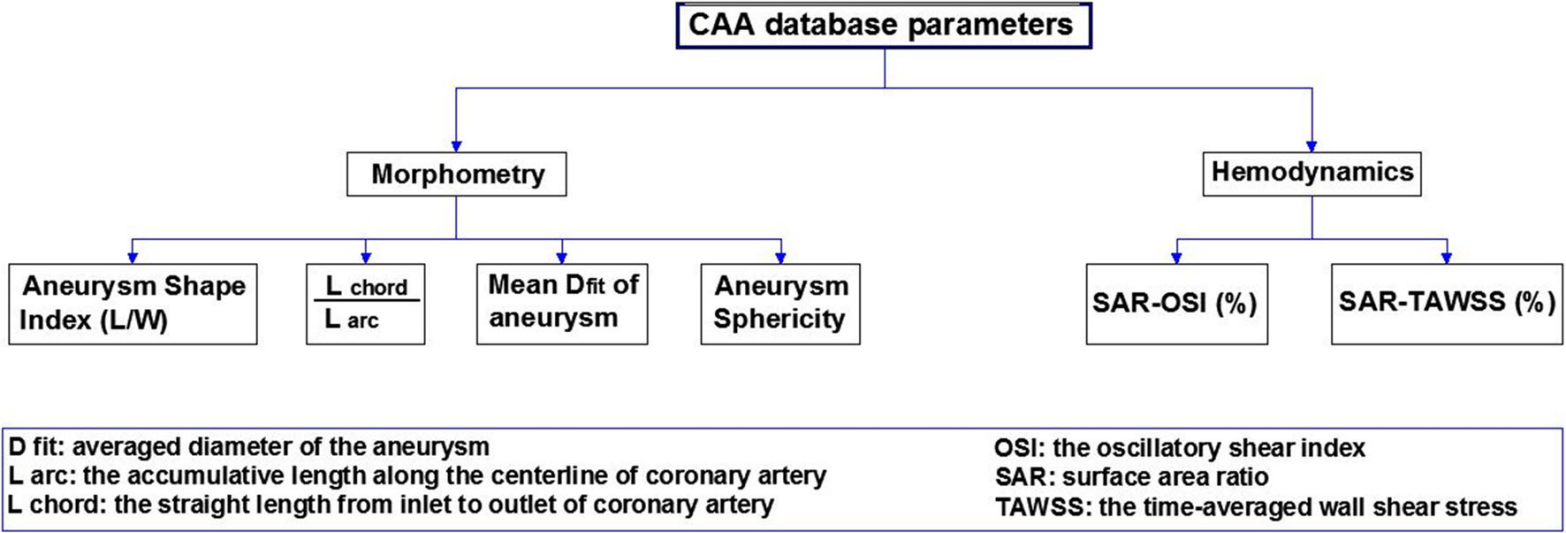
Database details from the morphometric and hemodynamic parameters of investigated CAAs

Approved patient-based data were provided by Fan et al. [30] for their study work in the hemodynamic and morphometric investigation of the 80 CAAs caused by atherosclerosis in 61 patients [29]. Figure 2 shows both morphometric and hemodynamic parameters presented in the dataset for atherosclerotic CAAs in epicardial coronary arteries considering the demographic features of the patients. The morphometric characteristics of CAAs were obtained from coronary CTA performed on three CT scanners. Three CT scanners of 256-row detector CT scanner [Revolution CT, GE Healthcare, Milwaukee, USA], 320–detector row [Aquilion One; Toshiba, Otawara, Japan], and dual-source [Somatom Definition Flash; Siemens, Forchheim, Germany] were used for preparing the coronary CT images from the patients. Then the recorded images were imported into the MIMICS Innovation Suite program (Materialise Company, Belgium) for the extraction of 3D geometries. Also, for hemodynamic parameters, Computational Fluid Dynamics (CFD) was carried out by ANSYS ICEM software (ANSYS Inc., Canonsburg, USA) based on morphometric features of the CAAs. For all samples, the Navier–Stokes and continuity equations were solved using a finite element method with applying the aortic pulsatile pressure as inlet and resistance boundary condition for each outlet during a cardiac cycle.

**Fig.3:**
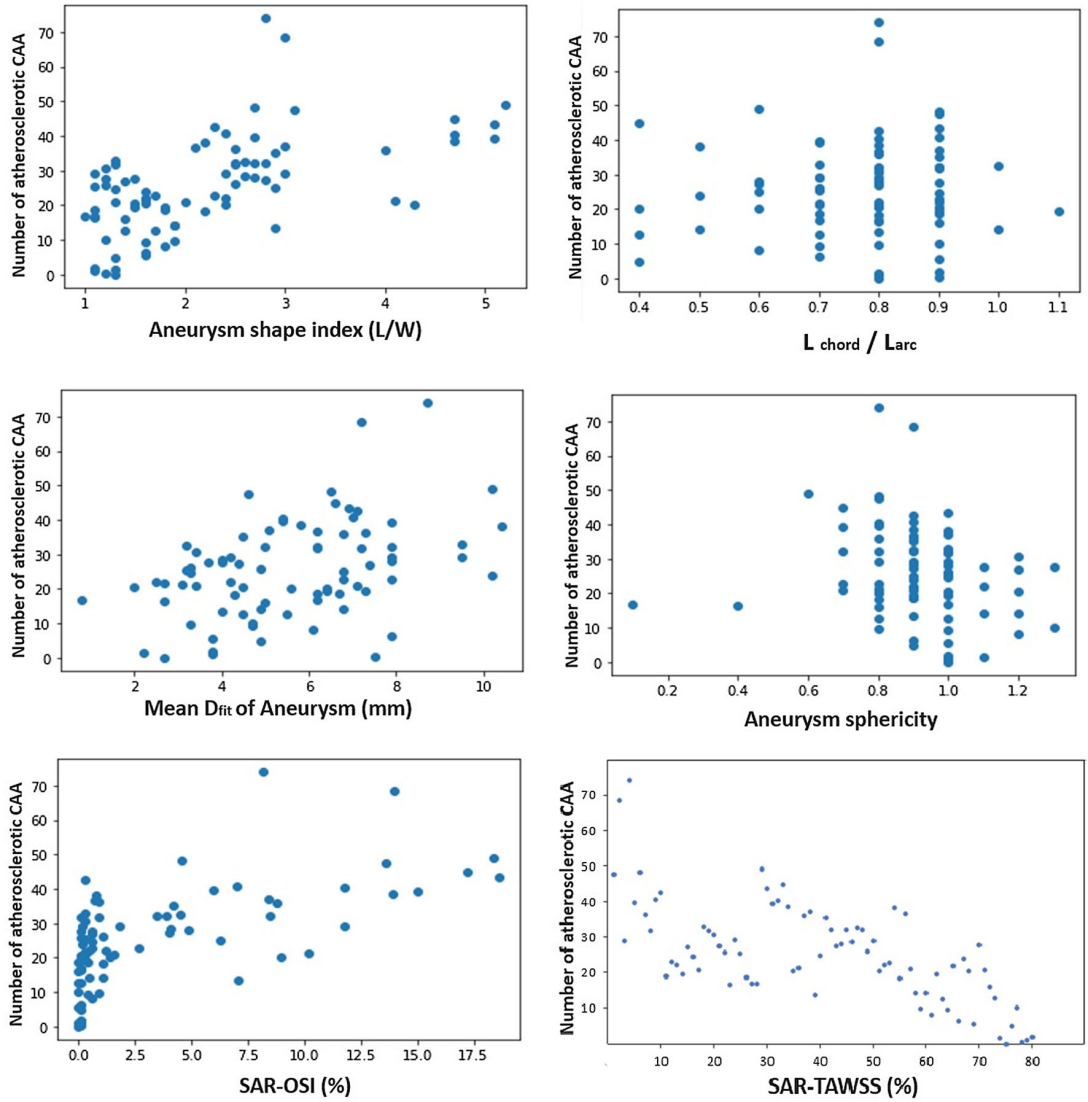
Distribution of the morphometric and hemodynamic features related to 80 CAAs recorded from the 61 patients. The aneurysm shape index (L/W) is a ratio resulted from the relationship between the maximum aneurysm diameter (W) and the aneurysm length (L). Using aneurysm volume (V) and surface area (A), the sphericity index (φ) can be calculated by $\varphi \; = \;\frac{{\pi^{1/3} . \left( {6V} \right)^{2/3} }}{A}$. The best fit diameter of the aneurysm (Mean D_fit_) is calculated in (mm) as twice the average radius between the point on the centerline and the contour of the 3D aneurysm vessel. And the straight length from inlet to outlet of coronary (L_chord_) and the accumulative length along the centerline of coronary artery (L_arc_) are needed for calculation of (L_chord_ /L_arc_). Besides, the hemodynamic parameter of surface area ratio of high oscillatory shear index (SAR-OSI) can be calculated by $\left( {\frac{{{\text{Surface area }}_{{{\text{OSI}} \ge 0.15}} }}{{\text{Aneurysmal surface area}}} \times 100} \right)$. Also, the formula $\left( {\frac{{{\text{Surface area}}_{{{\text{ TAWSS}} \le 0.4{ } dynes.{ }cm^{ - 2} { }}} }}{{\text{Aneurysmal surface area}}}{ } \times 100} \right)$ is used for calculation of surface area ratio of low time-averaged wall shear stress (TAWSS) where the (OSI ≥ 0.15) and $\left( {{\text{TAWSS}} \le 0.4{ }dynes.{ }cm^{ - 2} } \right){ }$ indicate the disease-prone site [30]

Statistical features of using dataset including all involved morphometric and hemodynamic variables are presented in Fig. 3. More details on both categories of the parameters are available on presented Supplementary Materials at the data article provided by Fan et al. [30]. The collected data were classified into four types according to the aneurysm shape index (L/W), where the L and W represent aneurysm length and maximum aneurysm diameter, respectively: (i) L/W ≥ 2 and CAA covering a bifurcation, (ii) L/W < 2 and CAA covering a bifurcation, (iii) L/W ≥ 2 and CAA in an artery, and (iv) L/W < 2 and CAA in an artery.

For designing the ML model, two features of the Oscillatory Shear Index (OSI) and the Time-Averaged Wall Shear Stress (TAWSS) according to the Surface Area Ratio (SAR) are selected as the hemodynamic effective parameters. OSI is a nondimensional hemodynamic parameter that indicates a strongly oscillating path during a pulse flow of cardiac cycle. Also for the morphological aspect, the four morphometric features of aneurysm shape index, (L_chord_ /L_arc_), aneurysm sphericity, and Mean D_fit_ are considered. Figures 2, 3 illustrate involving parameters in ML model designing and the statistical distributions of the parameters according to the database details. All of the atherosclerotic CAAs presented features in this dataset are already recorded and demonstrated by Fan et al. [30] for all 61 patients that were suffering from this lesion. Shortly, the machine needs all of the mentioned parameters to separate an atherosclerotic CAA from a non-atherosclerotic one with reflecting the ‘1’ as the final result after running. For entering the morphometric features, the patient-specific CTAs are needed but the hemodynamic-related data are taken from two-step CFD analysis of the involving artery and aneurysm.

### Data classification and ML algorithm

The working process of the proposed ML model depends on the mathematical principle of an algorithm that is able to classify variables to detection an unknown case by statistical analysis of the training set of normal cases. However, the learning of the ML model must be formulated by a one-class Support Vector Machine (1SVM) for anomaly detection. A one-class classification is a field of ML that provides techniques for outlier and anomaly detection [31]. In fact, the one-class classification algorithms can be used for classification tasks with a severely skewed class distribution. Also, the Support Vector Machine (SVM) is a computational learning method with significant applicability in estimating algorithms from a finite samples database. This learning method is based on Vapnik–Chervonenkis theory that is known as a self-consistent mathematical theory with a well-defined formulation feature. Furthermore, SVMs use hyperplanes in multi-dimensional space to separate one class of observations from another [32]. These techniques can be fit on the input examples from the majority class in the training dataset and then evaluated on a holdout test dataset. SVM is also increasingly being used in one-class problems, where all data belong to a single class. In this case, the algorithm is trained to learn what is “normal” so that when new data are shown, the algorithm can identify whether it should belong to the group or not. If not, the new data is labeled as out of ordinary or anomaly.

Therefore, 1SVM is a variation of the SVM that can be used in an unsupervised setting for anomaly detection. 1SVMs are the famous alternatives for designing unsupervised anomaly detection machines that can detect the basic distribution of normal data from the anomalies with evaluating in the training records. For obtaining an accurate and clear separation between normal and abnormal data, 1SVMs are supported by a kernel symmetric function that charts the input space to a higher-dimensional feature space. The Gaussian Radial Basis Function (RBF) kernel is the widely used kernel in 1SVMs. In this study, the RBF is used to assess the ability of 1SVM to afford the imbalanced datasets [33].

### One-class SVM for the ML model

**Fig. 4:**
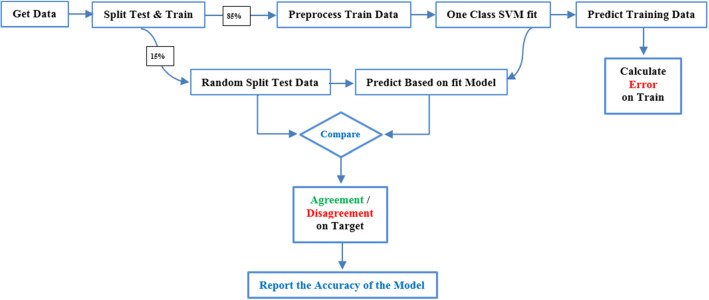
Architecture of the proposed SVM algorithm and its feasibility for detection of atherosclerotic aneurysm and the system accuracy based on morphometric and hemodynamic features

In situations where obtaining the one-class data are expensive or difficult, the one-class approach is the best choice for formulation of the ML model algorithms [33]. The preparing the atherosclerotic CAA data was also a big challenge in this study. Accordingly, the 1SVM is selected as a learning algorithm and method for the proposed ML model. In this study, the main goal is the detection of the atherosclerotic aneurysm for obtaining exact non-invasive information from the corresponding aneurysm for use in endovascular procedure and the selection of a suitable method. As a result, in this study, the aneurysm with atherosclerosis are formed as the majority class for two main reasons: the first is focusing on the diagnosis of a CAA that is into the which classes with or without atherosclerosis, and the second is the features of the existing datasets that all explain the atherosclerotic aneurysms. For this, because of having a data set of 80 patients all suffering from atherosclerotic CAAs, the information is targeted as (1). In other words, it is targeted the one class as (1) and any data that are given to this machine will be put in either class 1 (patient suffering from atherosclerotic CAA) or class (-1) (people suffering from non-atherosclerotic CAAs). Figure 4 shows the framework of the working principle of the 1SVM algorithm for the proposed model. All of 80 CAAs are diagnosed as atherosclerotic CAAs, and there is only one class for data classification wherein a binary output of the algorithm is required. Noteworthy, because of a limited data set, using a train-test split for splitting the existing data is not available. Hence, the more efficient technique for small data sets, the k-fold cross-validation splitting technique, is selected. The machine is designed to get data and separates them with 15–85 percent of variables to first for the random split test of the input data and second, for using in the preprocessing of the training step. The two learning paths proceed together and are joint in the prediction (based on fit model) step. The machine predicts the target for both training and testing data separately. Before comparing the results for these analyses, the probabilistic error on the train is calculated based on training data. In the comparison step, the machine tries to find a logical relationship between the normal training data and the split test data. Because of is a one-class algorithm, the results for split test and training must be correlated. In other words, the machine must be able to predict and classify the randomly selected input data as atherosclerotic CAA. The accuracy of the machine can be challenged by changing the split test and training data separation rate. Once the feasibility of the machine is demonstrated it can be employed for detection of the atherosclerotic and non-atherosclerotic CAAs based on six required features.

## Results and discussion on ML model feasibility

According to statistical information of Fig. 3, the majority of the CAAs are laid on the specific range in approximately all features. The selected samples that all are atherosclerotic CAAs, tend to classify into some ascertained range. Also, the aneurysm shape index for the database shows a maximal number of aneurysms from 1 to 3 including more than 50 samples. This range is limited to (0.6–0.9) for the (L_chord_ /L_arc_) feature. Although data distribution for the morphological feature of (Mean Df_it_) has not a specific range, graphical output of aneurysm sphericity shows that more of the CAAs have an (φ) index in the range of between 0.8 and 1. Besides, the distribution for hemodynamic parameters is different. Both OSI and TAWSS parameters display no regular pattern in feature distribution. But more than half of CAAs have an OSI in the range of (0–2.4), wherein a steady blood flow and intense oscillation (the disease-prone site within the CAA region) can be indicated by 0 and higher than 0.15 respectively [30]. Narrowing in arteries cross-section by atherosclerosis plaques aggregation leads to having turbulent blood flow. This causes significant changes in arterial wall shear stress and its relevant parameter of OSI. However, these data indicate that the defined indicators for the analysis during machine running are in the suitable range following medical scales for the morphology of the coronary arteries and their lesions considering the CFD analysis rules for demonstration of blood flowing through the aneurysmal regions.

**Fig. 5:**
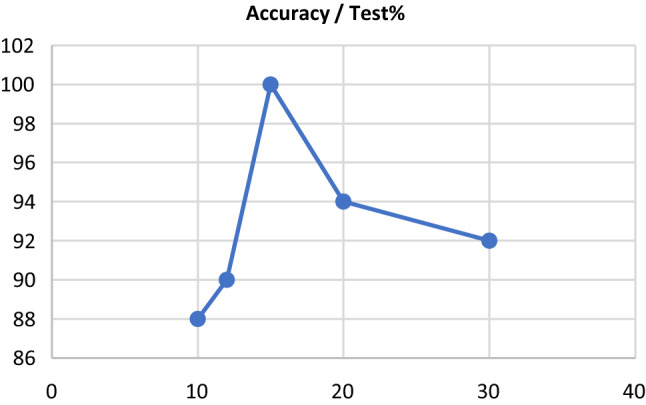
The machine accuracy according to applied test sets

For testing the feasibility of the proposed ML model, investigations are performed in the path like a flowchart illustrated in Fig. 4. The machine working starts with getting input data from the clinical information for both morphometric and hemodynamic parameters. With testing the randomly selected data in an SVM algorithm, the procedure continues and prediction based on the fit model helps comparing results and giving output data. The process ends with displaying and printing the predefined target output for detection of whether the corresponding CAA is atherosclerotic. Firstly, the data are divided into two categories of the test set and train set with a ratio of 15–85. By the use of a one-class SVMs algorithm, the train data set is processed with the prediction of the train data and calculation of its error rate. In the next step, the test data set is distributed randomly and then predicted based on the proposed algorithm with a Radial-Based Function (RBF) kernel. This function is supported by the (*nu*) parameter which is both a lower bound for the number of samples that are support vectors and an upper bound for the number of samples that are on the wrong side of the hyperplane. The nu parameter must be in the range [0,1], and the default is 0.5 which is used in this study. The output of these two-step trials is compared with each other numerically. As a first trial, the machine shows a 100% accuracy using a 15–85 ratio of the test set and train set. This precision declines to 88% if the mentioned ratio decreases to 10–90 (see Fig. 5). The fact that the reliability of health care relevant devices is a vital point in medical systems design was a determinative factor for studying the accuracy issue. The main cause of the decrease in accuracies is overfitting. Overfitting is the production of an analysis that corresponds too closely or exactly to a particular set of data and may therefore fail to fit additional data or predict future observations reliably. Also, overfitting is essentially learning spurious correlations that occur in training data and makes many problems especially when a small data set is used. In this case, it affected the accuracy percentage which was related to the split of the data in the training and test set.

Additionally, for the sensitivity, the model is tested with two main factors True Positive and False Negative. Mathematically, sensitivity can be calculated as (True Positive)/ (True Positive + False Negative) where true positive indicates samples predicted as suffering from the atherosclerotic CAA are actually suffering from the atherosclerotic CAA; in other words, the true positive represents the number of people who have at least an atherosclerotic CAA and are predicted as atherosclerotic CAA. Besides, false negative mentions to samples that are actually suffering from the atherosclerotic CAA are actually predicted to be not suffering from the atherosclerotic CAA. In other words, the false negative represents the number of people who have an atherosclerotic CAA and got predicted as having non-atherosclerotic CAA. Hence, the ideal model is to have low false negatives as it might prove to be life-threatening. In this study, a 10–90 ratio of test and train data gives a sensitivity of 87.5% (true positive = 7, false negative = 1, sensitivity = 7/8 = 0.875).

Consequently, the model demonstrates good capability in working for the non-invasive detection of atherosclerotic CAAs based on morphometric data taken from CTAs and hemodynamic features resulting from two-step CFD analysis. Hence, the placement of new data in the atherosclerotic or non-atherosclerotic CAA categories can be available based on these two important datasets. In other words, the machine is capable of detecting an atherosclerotic CAA by entering patient-specific recorded morphometric and hemodynamic features.

## Conclusions

Although atherosclerosis is one of the main causes of aneurysm formation with both biological and physiological dysfunctions, non-invasive detection of atherosclerotic aneurysms is vital before applying any treatment option. On the other hand, learning-based machines are pioneering statistical analysis and intelligent predictions in a variety of sectors. In this research, because of the non-invasive diagnosis importance of the atherosclerotic CAAs due to some clinical decisions and prevention of myocardial ischemia and infarction, an ML model was proposed. Prediction of the atherosclerotic CAAs using CTA-based morphometric data can decrease the probability of endovascular surgical failures, death risk, and post-operational complications. Also, it can increase the clinical decision accuracy for low-risk selection of treatment options. The feasibility of this machine was examined by good testing and accuracy results. Despite limitations in the preparation of CAA-related large datasets, a suitable ML algorithm of SVM was selected for analysis of the proven dataset. Due to lack of any ML approach on atherosclerotic CAA detection, it is expected that the proposed ML model could be a pioneering AI tool for not only the development of non-invasive diagnosis biomarkers but also to improve decision-making tools in clinical applications.

### Limitations

This study is performed having an approved patient-based data set related to 61 patients diagnosed with atherosclerotic aneurysms. In this case, all designing, testing, and verification procedure of the machine are laid on this limited data. On the other hand, due to some ethical issues and restrictions in sharing the medical data, accessibility to a data set containing patients recorded with non-atherosclerotic aneurysms was not possible. However, in order to obtain more reliable results, designing the machine with a high resolution in diagnosis, and developing a practical tool it is necessary to expand this study by considering both atherosclerotic and non-atherosclerotic aneurysms in future studies.

### Future work

**Fig. 6:**
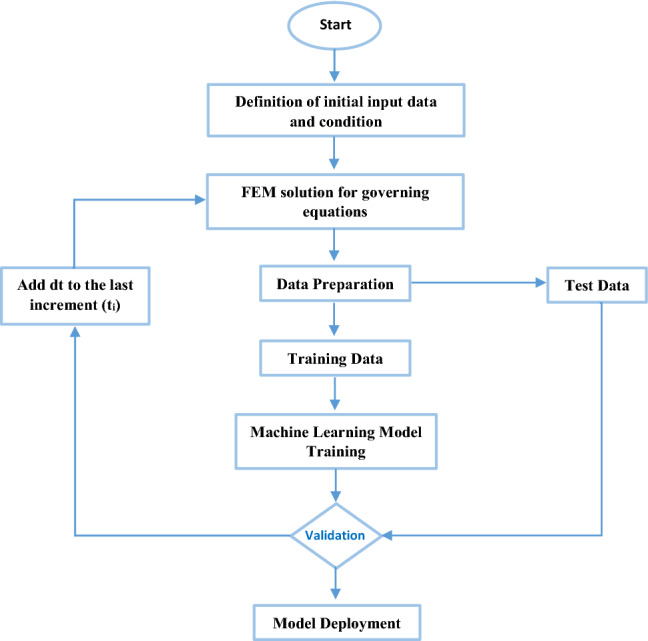
The future study path for using computational mechanics techniques to increase the accuracy and reliability of learning-based detection systems

Generally, computational models are developed within two main branches: mechanistic and statistical modeling. Mechanistic models assume some a priori knowledge of the behavior of physical processes but in the statistical method, sufficient data will aid in providing a mapping between input and output. Providing an adequate database for machine learning is a must to study in this field. In some cases, the achievement of experimental data is difficult as the study of the detection of atherosclerotic coronary. Finite Element Method (FEM) could play an important role to provide the necessary data for the machine. For illustration, Rostam-Alilou et al. [34] conducted a Fluid–Structure Interaction (FSI) study on the impact of atherosclerosis on hemodynamics, arterial tissue remodeling, and initiation risk of intracranial aneurysms. In future studies, the machine code could be linked to this study and the different parameters can be investigated in the FEM and the required data will be provided in this method. Figure 6 shows the future plan to extend the studies based on the mentioning idea.

## Data Availability

Not publicly available.
